# Pricing of medication abortion in the United States, 2021–2023

**DOI:** 10.1111/psrh.12280

**Published:** 2024-07-02

**Authors:** Ushma D. Upadhyay, Rosalyn Schroeder, Shelly Kaller, Clara Stewart, Nancy F. Berglas

**Affiliations:** ^1^ Advancing New Standards in Reproductive Health (ANSIRH), Department of Obstetrics, Gynecology, and Reproductive Sciences University of California San Francisco California USA

**Keywords:** abortion, costs, medication abortion, pricing, telehealth, telemedicine, United States

## Abstract

**Introduction:**

Financial costs remain one of the greatest barriers to abortion, leading to delays in care and preventing some from getting a desired abortion. Medication abortion is available through in‐person facilities and telehealth services. However, whether telehealth offers a more affordable option has not been well‐documented.

**Methods:**

We used Advancing New Standards in Reproductive Health (ANSIRH)'s Abortion Facility Database, which includes data on all publicly advertising abortion facilities and is updated annually. We describe facility out‐of‐pocket prices for medication abortion in 2021, 2022, and 2023, comparing in‐person and telehealth provided by brick‐and‐mortar and virtual clinics, and by whether states allowed Medicaid coverage for abortion.

**Results:**

The national median price for medication abortion remained consistent at $568 in 2021 and $563 in 2023. However, medications provided by virtual clinics were notably lower in price than in‐person care and this difference widened over time. The median cost of a medication abortion offered in‐person increased from $580 in 2021 to $600 by 2023, while the median price of a medication abortion offered by virtual clinics decreased from $239 in 2021 to $150 in 2023. Among virtual clinics, few (7%) accepted Medicaid. Median prices in states that accept Medicaid were generally higher than in states that did not.

**Discussion:**

Medication abortion is offered at substantially lower prices by virtual clinics. However, not being able to use Medicaid or other insurance may make telehealth cost‐prohibitive for some people, even if prices are lower. Additionally, many states do not allow telehealth for abortion, deepening inequities in healthcare.

## INTRODUCTION

Research on access to abortion consistently shows that, where abortion is legal, financial costs are the greatest barrier to obtaining this essential healthcare.^1^, ^2^, ^3^, ^4^, ^5^ These costs extend beyond paying for the abortion itself, and may also include transportation, gas, lodging, child care, and lost wages from time taken off work. These costs are compounded for people living in states that banned abortion after the U.S. Supreme Court's 2022 *Dobbs v. Jackson Women's Health Organization* decision, given that average travel distances for abortion care have increased significantly in the Midwest and the South.^6^ Together these costs lead to delays in care^1^ and may prevent some from getting a desired abortion altogether.^4^, ^7^

Awareness and use of medication abortion has increased in recent years.^8^, ^9^ Today it accounts for 63% of all abortions.^10^ Part of its increase is due to the rise of direct‐to‐patient no‐test telehealth a for abortion, which are medication abortions offered by a clinician through a remote consultation with the patient (via video, phone, or messaging) with medications dispensed by mail. Telehealth for abortion was introduced during the COVID‐19 pandemic and substantially reduced logistical burdens on patients.^11^, ^12^, ^13^, ^14^, ^15^, ^16^ Telehealth services may be offered through both brick‐and‐mortar abortion clinics as an alternative to in‐person care or through telehealth‐only virtual clinics, which have no physical/in‐person clinic space. As of March 2024, telehealth for abortion is available without restrictions in 24 states and Washington, DC.^16^, ^17^

After the *Dobbs* decision, telehealth services became vital to meeting increased demand for abortion by reducing appointment waiting times and serving people from states with abortion bans.^18^ Some people living in states with abortion bans have medications mailed to a location close to the border of a state where abortion is legal, or have medications mailed to a friend who forwards the medication to them in the banned state, to reduce the travel required for an in‐person visit.^19^

Additionally, between 2022 and 2024, 7 states passed shield laws that provide legal protections to clinicians in those states who offer abortion care to people living in states with abortion bans via telehealth. These states include Massachusetts, Colorado, Washington, New York, Vermont, California and Maine. As of December 2023, telehealth, including shield law abortions, accounted for 19% of all abortions in the U.S.^20^

Affordability of services is critical to abortion access^21^ as most patients pay out‐of‐pocket for abortion, usually because their health insurance does not cover it.^22^, ^23^ In a 2021 national survey of abortion patients, 60% reported paying out‐of‐pocket for their abortion.^23^ Among the 36 states where abortion remains legal, only 10 states require abortion coverage by private health insurance plans. In the remaining 26 states, insurance coverage is variable, dependent on the specific health insurance plan or the specific circumstances of the pregnancy, and patients must navigate through a complex set of rules to understand whether their abortion will be covered.^24^, ^25^

For people living on low‐incomes and relying on Medicaid for health insurance, only 17 states cover abortion.^11^ Even when Medicaid covers abortion, it may not cover telehealth abortions. Medicaid coverage of telehealth abortion varies widely from state to state, due to the wide latitude states maintain in determining telehealth coverage, including defining what constitutes as telehealth, which providers and services are eligible for reimbursement, and developing reimbursement structures.^26^

Given that telehealth is a relatively new addition to the abortion care landscape, and because telehealth services have expanded dramatically, we report on prices of medication abortion, both in‐person and via telehealth. We report mean and median prices of out‐of‐pocket costs a patient would have to pay without insurance coverage or other funding. For telehealth services, we report prices of virtual clinics compared to brick‐and‐mortar telehealth services. We present prices in 2021, 2022, and 2023 to examine changes in the price of medication abortion before and after the *Dobbs* decision.

## METHODS

### Data collection

For this analysis we used data from the Advancing New Standards in Reproductive Health (ANSIRH) Abortion Facility Database, which includes data on all publicly advertising abortion facilities and is systematically updated from May to September every year. The database includes a wide range of facilities, including doctor's offices, public health centers, and hospital settings. We updated the database annually using a systematic process of online searches to identify abortion facilities followed by mystery shopper calls to confirm and obtain additional information from the facilities when a phone number was available. Each year we confirmed whether each facility in the database was still open, and for those open, we updated variables on pricing, insurance acceptance, and other data. We also added any facilities that had newly begun to offer abortion care. When possible, we cross‐checked the list against additional abortion provider directories, including ineedana.com and www.abortionfinder.org, as well as abortion facility organizational membership lists. More detailed information on our data collection methodology can be found in our previous paper on out‐of‐pocket prices for abortion.^27^ The University of California, San Francisco's Institutional Review Board approved the study.

Through these searches and calls, we collected data on whether the clinic offered medication abortion services, whether they offered telehealth for abortion with medications delivered by mail, and the self‐pay charges for medication abortion. Beginning in 2023, we began to ask brick‐and‐mortar clinics that offered telehealth whether they had different prices for in‐person and telehealth care. In 2023, we also began to add telehealth services operating within the U.S. healthcare system regardless of whether they provided care into states that legally permit telehealth for abortion. Thus, Aid Access and Abuzz, which began to provide care under shield laws into states with abortion bans or restrictions on telehealth abortion in 2023, were included as of that year.^28^ All telehealth services were counted in the states they mailed medications to (not the states where they prescribed from).

Virtual clinics that served multiple states were counted as separate facilities, one for each state in which it serves. Thus, if a virtual clinic operated in 20 states, it was counted as 20 separate facilities. In this way, we were able to capture prices or insurance policies that may have differed by state, even when offered by the same virtual clinic. Most data for virtual clinics could not be verified by a mystery shopper call, so we relied on information listed on their website.

Brick‐and‐mortar facilities in the same state that were part of the same affiliate or facility group were counted as a single telehealth provider because they advertised their telehealth services jointly, including a common website or phone number, and common pricing. We refer to these as “facility groups.” When facility groups spanned more than one state, they were counted separately by state.

### Data analysis

For each year, we report the mean and median prices for medication abortion services by state, subregion, region, and nationally. However, for subsequent analyses we primarily report medians due to the non‐normal distribution of abortion price data and to reduce the impact of outliers. We then describe median prices for in‐person services and virtual clinics. We also describe the proportion of facilities nationally that accept Medicaid by whether they offer telehealth services. Finally, we describe median pricing among states that allow for Medicaid coverage of medication abortion and states that do not.

For 2023, telehealth prices are disaggregated by brick‐and‐mortar and virtual services where data were available. Because many brick‐and‐mortar facilities indicated separate prices for in‐person versus telehealth medication abortion services, we assumed that the price of telehealth services was the same as in‐person medication abortion services when separate prices were not provided.

Facilities were included in the analysis if they reported being open and providing medication abortions in a given year. For telehealth, prices represent the price for patients by their state of residence, not the state that the clinicians were prescribing from. To compute prices for facilities that gave a range of prices for medication abortion services, we first calculated a mean price per facility. All analyses were completed using Stata 17.

## RESULTS

We identified 773 facilities that were open and providing medication abortion services in 2021, 789 in 2022, and 961 in 2023. We identified 31 virtual clinics in 2021 (4% of all facilities), 69 in 2022 (9% of all facilities) and 226 in 2023 (24% of all facilities). We obtained medication abortion pricing information from 748 facilities (97%) in 2021, 725 facilities (92%) in 2022, and 941 facilities (98%) in 2023.

Between 2021 and 2023, the national median medication abortion price decreased slightly from $568 in 2021 to $563 in 2023. In 2023, prices were highest in the West region at $612 and lowest in the Midwest at $475 (Table 1). Hospitals listed the highest prices for medication abortion, frequently over $1000.

**TABLE 1:** Mean and median self‐pay prices of medication abortion services (in USD$), stratified by state and geographic region, 2021–2023.

	2021		2022		2023	
*Geographic region and state*	Mean Cost among all Facilities (*n* = 748)	Median Cost among all Facilities (*n* = 748)	Mean Cost among all Facilities (*n* = 725)	Median Cost among all Facilities (*n* = 725)	Mean Cost among all Facilities (*n* = 941)	Median Cost among all Facilities (*n* = 941)
**United States (Total)**	**658** **(150–6300)**	**568** **(150–6300)**	**648** **(145–6300)**	**560** **(145–6300)**	**570** **(113–6000)**	**563** **(113–6000)**
**Northeast**	**610** **(239–6300)**	**550** **(239–6300)**	**581** **(145–6300)**	**550** **(145–6300)**	**495** **(113–2100)**	**550** **(113–2100)**
**New England**	**722**	**555**	**609**	**555**	**456**	**525**
Connecticut	1220	620	868	619	486	600
Maine	491	500	477	500	443	525
Massachusetts	679	650	640	650	546	650
New Hampshire	525	555	556	586	435	553
Rhode Island	530	600	434	420	309	200
Vermont	510	555	440	555	353	264
**Middle Atlantic**	**551**	**550**	**565**	**550**	**521**	**555**
New Jersey	463	490	460	490	416	483
New York	612	580	637	600	580	600
Pennsylvania	466	448	484	500	503	555
**Midwest**	**572** **(239–834)**	**550** **(239–834)**	**569** **(145–834)**	**550** **(145–834)**	**488** **(113–1000)**	**475** **(113–1000)**
**East North Central**	**543**	**550**	**542**	**513**	**495**	**475**
Illinois	451	470	457	470	441	470
Indiana	773	834	802	834	150‡	150‡
Michigan	537	550	538	550	558	600
Ohio	637	650	709	700	633	650
Wisconsin	619	600	*	*	150‡	150‡
**West North Central**	**656**	**730**	**650**	**730**	**476**	**560**
Iowa	673	730	673	730	542	730
Kansas	714	735	739	735	559	743
Minnesota	596	650	577	603	484	400
Missouri	†	†	*	*	150‡	150‡
Nebraska	720	730	720	730	512	730
North Dakota	650	650	650	650	333‡	150‡
South Dakota	661	661	*	*	150‡	150‡
**South**	**537** **(239‐1200)**	**520** **(239–1200)**	**516** **(145–3500)**	**493** **(145–3500)**	**491** **(125–6000)**	**500** **(125–6000)**
**South Atlantic**	**505**	**495**	**516**	**493**	**510**	**500**
Delaware	406	490	381	490	315	269
District of Columbia	557	425	301	282	389	237
Florida	529	546	540	550	575	565
Georgia	460	500	460	500	486	500
Maryland	420	390	565	400	580	450
North Carolina	671	425	671	425	538	600
South Carolina	528	495	528	495	461	625
Virginia	424	450	431	450	420	450
West Virginia	495	495	*	*	150‡	150‡
**East South Central**	**626**	**600**	*	*	**150** ‡	**150** ‡
Alabama	600	600	*	*	150‡	150‡
Kentucky	767	767	*	*	150‡	150‡
Mississippi	600	600	*	*	150‡	150‡
Tennessee	607	600	*	*	150‡	150‡
**West South Central**	**649**	**650**	*	*	**150** ‡	**150** ‡
Arkansas	722	722	*	*	150‡	150‡
Louisiana	567	600	*	*	150‡	150‡
Oklahoma	673	650	*	*	150‡	150‡
Texas	651	700	*	*	150‡	150‡
**West**	**805** **(150‐3000)**	**650** **(150–3000)**	**792** **(145–2500)**	**669** **(145–2500)**	**700** **(140–2500)**	**612** **(140–2500)**
**Mountain**	**583**	**550**	**561**	**560**	**454**	**555**
Arizona	559	540	609	570	622	720
Colorado	588	458	633	560	484	600
Idaho	649	650	*	*	150‡	150‡
Montana	533	555	470	555	378	350
Nevada	675	600	579	600	482	600
New Mexico	518	560	496	560	427	470
Utah	450	450	450	450	408	525
Wyoming	600	600	475	475	370	350
**Pacific**	**868**	**700**	**856**	**700**	**794**	**650**
Alaska	700	800	675	800	540	800
California	927	700	931	700	890	612
Hawaii	675	850	675	850	391	293
Oregon	603	650	570	600	551	700
Washington	722	650	626	650	558	650

*Note*: Rows shown in dark pink are regional values. Rows shown in light grey are subregional values. Rows shown in white and peach are state values.

* Abortion was banned in the state and this study did not collect data on providers operating outside the U.S. healthcare system.

^†^ Missouri's sole clinic did not provide medication abortion services in 2021.

^‡^ One or more telehealth providers operated under state shield laws to offer abortion care in states with total abortion bans or restrictions on telehealth abortion.

**FIGURE 1:**
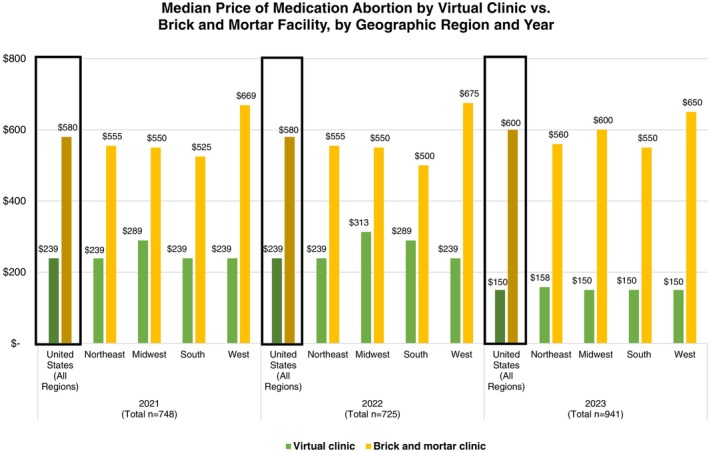
Median price of medication abortion by facility type, geographic region, and by year of data collection.

Telehealth was generally lower in price than in‐person care. The median cost of a medication abortion offered in‐person *increased* from $580 in 2021 to $600 by 2023 (Table 2 and Figure 1). The median cost of a medication abortion offered by virtual clinics *decreased* from $239 in 2021 to $150 in 2023. In 2023, the year we began to collect pricing data on brick‐and‐mortar clinics that offered telehealth care, 51 facilities/facility groups nationwide offered both in‐person and telehealth care. Among these brick‐and‐mortar facilities/facility groups, 37% (*n* = 19) advertised lower prices for in‐person and telehealth care while the rest offered both at the same price. The median price for in‐person care was $600 while the median price for telehealth was $500 (Table 2).

**TABLE 2:** Median self‐pay prices of medication abortion services (in USD$) by telehealth services versus in‐person services, stratified by state and geographic region, 2021–2023.

	2021		2022		2023		
*Geographic region and state*	Median Cost among Brick and Mortar Facilities (All services^; *n* = 717)	Median Cost among Virtual Clinics (Telehealth services only; *n* = 31)	Median Cost among Brick and Mortar Facilities (All services^; *n* = 665)	Median Cost among Virtual Clinics (Telehealth services only; *n* = 60)	Median Cost among Brick and Mortar Facilities (In‐person services; *n* = 721)	Median Cost Among Brick and Mortar Facilities/Facility Groups (Telehealth services; *n* = 51)	Median Cost among Virtual Clinics (Telehealth services only; *n* = 226)
**United States (Total)**	**580**	**239**	**580**	**239**	**600**	**500**	**150**
**Northeast**	**555**	**239**	**555**	**239**	**560**	**638**	**158**
**New England**	**578**	**239**	**600**	**239**	**600**	**650**	**158**
Connecticut	620	276	619	289	600	521	200
Maine	500	239	500	192	525	525	182
Massachusetts	650	239	650	192	700	675	200
New Hampshire	555	239	650	239	603	#	150
Rhode Island	675	239	675	192	675	#	150
Vermont	555	239	555	289	555	#	200
**Middle Atlantic**	**550**	**244**	**550**	**289**	**560**	**625**	**175**
New Jersey	490	276	490	289	490	325	175
New York	580	244	600	289	600	700	200
Pennsylvania	448	¶	500	¶	625	625	150
**Midwest**	**550**	**289**	**550**	**313**	**600**	**483**	**150**
**East North Central**	**550**	**264**	**550**	**289**	**600**	**495**	**150**
Illinois	470	264	478	289	470	482	245
Indiana	834	§	834	§	*	*	150‡
Michigan	550	¶	550	¶	600	600	150
Ohio	650	¶	700	¶	700	¶	150
Wisconsin	600	§	*	*	*	*	150‡
**West North Central**	**730**	**313**	**730**	**331**	**733**	**425**	**150**
Iowa	730	313	730	313	730	p	150
Kansas	735	§	735	§	750	§	150‡
Minnesota	793	295	793	350	861	425	150
Missouri	†	†	*	*	*	*	150‡
Nebraska	730	§	730	§	730	§	150‡
North Dakota	650	§	*	*	*	*	150‡
South Dakota	661	§	*	*	*	*	150‡
**South**	**525**	**239**	**500**	**289**	**550**	**400**	**150**
**South Atlantic**	**495**	**239**	**500**	**289**	**550**	**400**	**150**
Delaware	490	239	490	217	500	#	158
District of Columbia	475	239	320	217	495	372	150
Florida	546	¶	550	¶	565	¶	150‡
Georgia	500	239	500	276	500	#	150
Maryland	390	239	400	390	500	450	175
North Carolina	425	§	425	§	600	§	150‡
South Carolina	495	§	495	§	625	§	150‡
Virginia	450	276	468	239	475	400	220
West Virginia	495	§	*	*	*	*	150‡
**East South Central**	**600**	§	*	*	*	*	**150** ‡
Alabama	600	§	*	*	*	*	150‡
Kentucky	767	§	*	*	*	*	150‡
Mississippi	600	§	*	*	*	*	150‡
Tennessee	600	§	*	*	*	*	150‡
**West South Central**	**650**	§	*	*	*	*	**150** ‡
Arkansas	722	§	*	*	*	*	150‡
Louisiana	600	§	*	*	*	*	150‡
Oklahoma	650	§	*	*	*	*	150‡
Texas	700	§	*	*	*	*	150‡
**West**	**669**	**239**	**675**	**239**	**650**	**500**	**150**
**Mountain**	**555**	**239**	**560**	**239**	**625**	**500**	**150**
Arizona	540	§	570	§	750	§	150‡
Colorado	458	239	560	217	625	500	199
Idaho	650	¶	*	*	*	*	150‡
Montana	555	239	555	239	555	300	150
Nevada	600	239	600	239	613	625	249
New Mexico	560	239	580	192	625	500	200
Utah	450	¶	450	¶	550	#	150
Wyoming	600	¶	600	350	600	#	150
**Pacific**	**700**	**239**	**700**	**219**	**700**	**500**	**175**
Alaska	800	¶	800	¶	800	#	150
California	700	234	700	210	675	500	223
Hawaii	850	¶	850	¶	850	#	150
Oregon	675	239	675	192	700	5500	150
Washington	650	244	650	239	650	502	200

*Note*: Rows shown in dark pink are regional values. Rows shown in light grey are subregional values. Rows shown in white and peach are state values.

^^^ Estimates may include both in‐person and telehealth medication abortion services.

* Abortion was banned in the state and this study did not collect data on providers operating outside the U.S. healthcare system.

^†^ Missouri's sole clinic did not provide medication abortion services in 2021.

^‡^ One or more telehealth providers operated under state shield laws to offer abortion care in states with total abortion bans or restrictions on telehealth abortion.

^§^ Telehealth for medication abortion prohibited in the state.

^¶^ No virtual/telehealth facilities operating at time of data collection.

^#^ No brick and mortar facilities providing telehealth services at the time of data collection.

**FIGURE 2:**
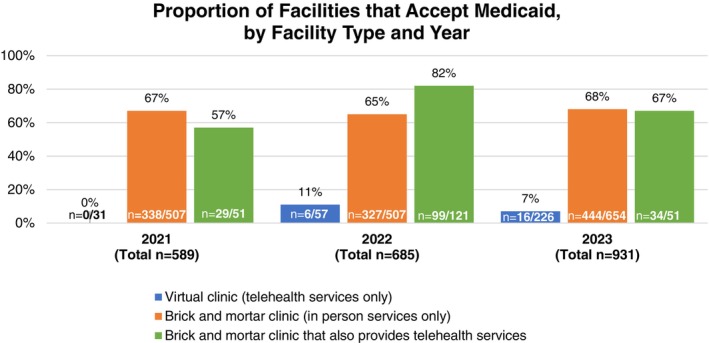
Proportion of facilities that accept Medicaid insurance, by facility type and year of data collection.

Most virtual clinics did not accept Medicaid. In 2021, none of the 31 (0%) virtual clinics accepted Medicaid, increasing to 16 out of 226 (7%) in 2023 (Figure 2). However, among the 51 brick‐and‐mortar facilities/facility groups that also offered telehealth services in 2023, 34 (67%) accepted Medicaid.

**FIGURE 3:**
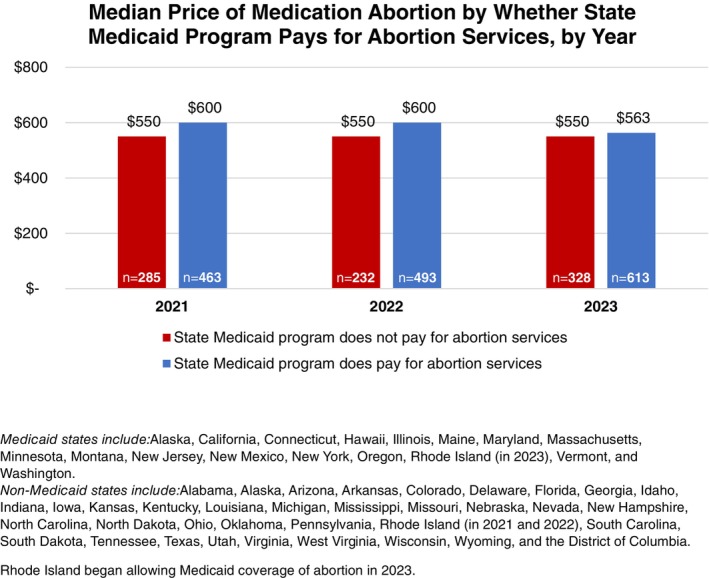
Median price of medication abortion in states that allow Medicaid coverage versus non‐Medicaid coverage states, by year of data collection. Medicaid states include: Alaska, California, Connecticut, Hawaii, Illinois, Maine, Maryland, Massachusetts, Minnesota, Montana, New Jersey, New Mexico, New York, Oregon, Rhode Island (in 2023), Vermont, and Washington. Non‐Medicaid states include: Alabama, Alaska, Arizona, Arkansas, Colorado, Delaware, Florida, Georgia, Idaho, Indiana, Iowa, Kansas, Kentucky, Louisiana, Michigan, Mississippi, Missouri, Nebraska, Nevada, New Hampshire, North Carolina, North Dakota, Ohio, Oklahoma, Pennsylvania, Rhode Island (in 2021 and 2022), South Carolina, South Dakota, Tennessee, Texas, Utah, Virginia, West Virginia, Wisconsin, Wyoming, and the District of Columbia. Rhode Island began allowing Medicaid coverage of abortion in 2023.

Median prices for medication abortion in states that allow Medicaid coverage for abortion were higher than in states that did not allow Medicaid coverage in 2021 and 2022 ($600 for Medicaid‐accepting states and $550 for non‐Medicaid states for both years, *p* < 0.001). However, in 2023, median costs were similar ($563 in Medicaid‐accepting states vs. $550 in non‐Medicaid states, *p* = not significant) (Figure 3, Table 3).

**TABLE 3:** Median self‐pay prices of medication abortion service costs (in USD$) by whether state allows for state Medicaid coverage of abortion services, stratified by state and geographic region, 2021–2023.

	2021		2022		2023	
Geographic region and state	Median Cost among Facilities in States that Allow for Medicaid Coverage of Abortion Services (*n* = 463)	Median Cost among Facilities in States that Do Not Allow for Medicaid Coverage of Abortion Services (*n* = 285)	Median Cost among Facilities in States that Allow for Medicaid Coverage of Abortion Services (*n* = 493)	Median Cost among Facilities in States that Do Not Allow for Medicaid Coverage of Abortion Services (*n* = 232)	Median Cost among Facilities in States that Allow for Medicaid Coverage of Abortion Services (*n* = 613)	Median Cost among Facilities in States that Do Not Allow for Medicaid Coverage of Abortion Services (*n* = 328)
**United States (Total)**	**600**	**550**	**600**	**550**	**563**	**550**
**Northeast**	**555**	**500**	**555**	**500**	**525**	**553**
**New England**	**555**	**555**	**555**	**561**	**525**	**553**
Connecticut	620	§	619	§	600	§
Maine	500	§	500	§	525	§
Massachusetts	650	§	650	§	650	§
New Hampshire	¶	555	¶	586	¶	553
Rhode Island	¶	600	¶	420	200	§
Vermont	555	§	555	§	264	§
**Middle Atlantic**	**555**	**448**	**555**	**500**	**555**	**555**
New Jersey	490	§	490	§	483	§
New York	580	§	600	§	600	§
Pennsylvania	¶	448	¶	500	¶	555
**Midwest**	**470**	**638**	**475**	**650**	**470**	**600**
**East North Central**	**470**	**551**	**470**	**550**	**470**	**600**
Illinois	470	§	470	§	470	§
Indiana	¶	834	¶	834	¶	150‡
Michigan	¶	550	¶	550	¶	600
Ohio	¶	650	¶	700	¶	650
Wisconsin	¶	600	¶	*	¶	150‡
**West North Central**	**650**	**730**	**603**	**730**	**400**	**600**
Iowa	¶	730	¶	730	¶	730
Kansas	¶	735	¶	735	¶	743
Minnesota	650	§	603	§	400	§
Missouri	†	†	¶	*	¶	150‡
Nebraska	¶	730	¶	730	¶	730
North Dakota	¶	650	¶	*	¶	150‡
South Dakota	¶	661	¶	*	¶	150‡
**South**	**390**	**543**	**400**	**498**	**450**	**500**
**South Atlantic**	**390**	**500**	**400**	**498**	**450**	**525**
Delaware	¶	490	¶	490	¶	269
District of Columbia	¶	425	¶	282	¶	237
Florida	¶	546	¶	550	¶	565
Georgia	¶	500	¶	500	¶	500
Maryland	390	§	400	§	450	§
North Carolina	¶	425	¶	425	¶	600
South Carolina	¶	495	¶	495	¶	625
Virginia	¶	450	¶	450	¶	450
West Virginia	¶	495	¶	*	¶	150‡
**East South Central**	¶	**600**	¶	*	¶	**150** *
Alabama	¶	600	¶	*	¶	150‡
Kentucky	¶	767	¶	*	¶	150‡
Mississippi	¶	600	¶	*	¶	150‡
Tennessee	¶	600	¶	*	¶	150‡
**West South Central**	¶	**650**	¶	*	¶	**150** ‡
Arkansas	¶	722	¶	*	¶	150‡
Louisiana	¶	600	¶	*	¶	150‡
Oklahoma	¶	650	¶	*	¶	150‡
Texas	¶	700	¶	*	¶	150‡
**West**	**678**	**510**	**676**	**560**	**612**	**600**
**Mountain**	**555**	**510**	**555**	**560**	**379**	**600**
Arizona	¶	540	¶	570	¶	720
Colorado	¶	458	¶	560	¶	600
Idaho	¶	650	¶	*	¶	150‡
Montana	555	§	555	§	350	§
Nevada	¶	600	¶	600	¶	600
New Mexico	560	§	560	§	470	§
Utah	¶	450	¶	450	¶	525
Wyoming	¶	600	¶	475	¶	350
**Pacific**	**700**	§	**700**	§	**650**	§
Alaska	800	§	800	§	800	§
California	700	§	700	§	612	§
Hawaii	850	§	850	§	293	§
Oregon	650	§	600	§	700	§
Washington	650	§	650	§	650	§

*Note*: Rows shown in dark pink are regional values. Rows shown in light grey are subregional values. Rows shown in white and peach are state values.

* Abortion was banned in the state and this study did not collect data on providers operating outside the U.S. healthcare system.

^†^ Missouri's sole clinic did not provide medication abortion services in 2021.

^‡^ One or more telehealth providers operated under state shield laws to offer abortion care in states with total abortion bans or restrictions on telehealth abortion.

^§^ State Medicaid program allows for coverage of abortion services.

^¶^ State Medicaid program does not allow for coverage of abortion services.

## DISCUSSION

This study finds that telehealth for abortion, particularly provided by virtual clinics, can greatly reduce medication abortion costs for patients. We observed a rapid increase in the number of virtual clinics from 2021 to 2023, accounting for almost a quarter of all abortion providers by 2023. We also found that in all states with bans on abortion or restrictions on telehealth abortion, the median cost for medication abortion services was $150, lower than in states that allow abortion and telehealth for abortion, which is driven by the lower cost of services among providers working under shield laws.

Because telehealth typically involves omitting pre‐abortion ultrasounds and/or other tests and does not require a physical space, thus reducing operational costs, virtual clinics can offer medication abortion at a lower price than in‐person facilities^29^, ^30^ Additionally, providers operating under shield laws charge the lowest prices, just covering their costs.^28^ In‐person abortion care will always be essential, as some people prefer to see a provider in person, others require ultrasounds or other in‐person tests to confirm eligibility for medication abortion, and still others prefer or need procedural abortions. But given that nationally, three‐quarters of abortion patients have low incomes,^23^ telehealth's low price point could be the critical difference between having and not having an abortion for many people.

We found some variation by region, with the highest prices for medication abortion in the West and the lowest in the Midwest. While our data do not allow us to make conclusions about reasons for differences in price, we speculate that regional variations are due to differences in staff pay, property, and other costs.

Depending on the state they live in, some patients may be able to use their health insurance to cover their abortion. However, given that most virtual clinics do not accept insurance including Medicaid, patients in these states may feel compelled to get in‐person services. Thus, they may not feel they have the option to get a telehealth abortion. We also found that the out‐of‐pocket price, on average, has been historically higher in states that cover abortion costs through Medicaid than in states that do not. Previous studies suggest this is due to attempts to compensate for low reimbursement rates from their state Medicaid programs.^34^ Thus, patients in Medicaid states who seek medication abortion may have to pay higher out‐of‐pocket prices if their insurance does not cover it.^31^

This study fills a gap, providing needed state, subregional, regional, and national facility pricing estimates for medication abortion. A major strength of the study is the completeness of data given the systematic census approach to data collection, thus improving generalizability. One limitation is that for 2021 and 2022, we did not have pricing for brick‐and‐mortar facilities that disaggregate prices between in‐person and telehealth services. When we collected disaggregated prices for 2023, we found that 37% of clinics had different prices for in‐person versus telehealth services, which were always lower for telehealth. Another limitation is that we used only a single price per clinic, even though many clinics offer patients sliding scale fees. Some even offer services at no charge for people who cannot afford to pay anything.^28^ Others offer immediate funding from an abortion fund. Our methods did not allow us to use price ranges; instead we used the price that was advertised on the website or reported to our staff as the price of the abortion. A final limitation is that while additional new providers began to offer services under shield laws to patients in states with abortion bans at the end of 2023, our methods may not have included them if they began services after the end of our data collection period.

Medication abortion is preferred by many people seeking abortion, yet costs remain high and variable. In‐person care will always be preferred by some patients and needed for patients who are not medically eligible for a no‐test medication abortion. However, telehealth services—and particularly virtual clinics—offer the potential to reduce costs for those who do not have access to or do not want to use their health insurance for abortion care. Thus, telehealth may increase health equity in abortion access. This is consistent with previous research findings that telehealth makes the difference in obtaining timely abortion care for marginalized groups, such as younger people, people who experience food‐insecurity, and people living in rural areas.^32^, ^33^ Increasing affordability is critical to abortion access.^21^ It is vital that abortion care be low‐cost or no‐cost and accessible, especially as legal barriers to abortion increase.

## Footnotes

^a^
In this paper we use the term telehealth because that is the term used by the vast majority of the facilities offering such services. 
